# The Pivotal Role of LACTB in the Process of Cancer Development

**DOI:** 10.3390/ijms26031279

**Published:** 2025-02-01

**Authors:** Minghui Zhang, Bowen Wu, Jinke Gu

**Affiliations:** 1 Guangdong Key Laboratory of Genome Instability and Human Disease Prevention, Department of Biochemistry and Molecular Biology, School of Basic Medical Sciences, Shenzhen University Medical School, Shenzhen 518055, China; zhangmh@szu.edu.cn (M.Z.); wubowen@szu.edu.cn (B.W.); 2 Guangdong Key Laboratory for Biomedical Measurements and Ultrasound Imaging, National-Regional Key Technology Engineering Laboratory for Medical Ultrasound, School of Biomedical Engineering, Shenzhen University Medical School, Shenzhen 518060, China

**Keywords:** LACTB, cancer research, tumor suppression, oncogene, structure-function relationship, therapeutic target

## Abstract

The mitochondrial serine β-lactamase-like protein LACTB has emerged as a critical regulator in cancer biology, distinguished by its unique structural and functional attributes. Defined by its conserved penicillin-binding proteins and β-lactamases (PBP-βLs) domain and SXXK catalytic motif, LACTB demonstrates properties distinct from its prokaryotic homologs, including the ability to polymerize into filaments. These structural characteristics enable LACTB to modulate mitochondrial organization and enzymatic activity, influencing lipid metabolism and indirectly affecting cellular proliferation. Importantly, the expression and functional roles of LACTB exhibit cancer-type-specific variation, underscoring its dual function as both a tumor suppressor and an oncogene. Decreased LACTB expression is associated with poor clinical outcomes in cancers such as breast cancer, lung cancer, and colorectal cancer, while specific mutations and regulatory mechanisms have been linked to its oncogenic activity in osteosarcoma and pancreatic adenocarcinoma. Mechanistically, LACTB regulates key processes in cancer progression, including mitochondrial dynamics, epithelial–mesenchymal transition (EMT), and cell death pathways. This duality highlights LACTB as a promising therapeutic target and underscores its relevance in advancing precision oncology strategies. This review provides a comprehensive analysis of expression level, structure–function relationships, and the diverse roles of LACTB in oncogenesis, underscoring its promise as a focal point for precision cancer therapies.

## 1. Introduction

The serine beta-lactamase-like protein LACTB is a mammalian mitochondrial serine proteinase recognized for hydrolyzing peptide bonds following aspartic acid residues [1]. Beyond its signal peptide, LACTB contains conserved penicillin-binding proteins and β-lactamases (PBP-βLs) domain, which shares high homology with the prokaryotic PBP-βLs family, and features a distinct middle region. Unlike its prokaryotic homologs, LACTB polymerizes into long filaments, a structural feature believed to facilitate mitochondrial membrane organization and micro-compartmentalization [2,3]. This filamentous formation significantly influences both the enzymatic activity of LACTB and its membrane interactions.

LACTB is involved in several critical biological functions, including lipid metabolism, immune response, regulation of obesity, modulation of atherosclerosis, and skeletal muscle formation and regeneration [4,5,6,7,8]. Beyond these fundamental roles, recent studies have emphasized its emerging significance in cancer. Altered LACTB expression and mutations are strongly linked to cancer progression and poor prognosis. In light of the differential regulation of LACTB in cancers, along with its unique tumor-suppressive or oncogenic functions, targeting LACTB represents an impending therapeutic strategy in precision medicine.

Cancer remains one of the most significant global public health challenges today [9]. With changes in lifestyle patterns, the increasing impact of environmental factors, and an aging population, the global cancer burden continues to escalate. In light of its pivotal roles in cancer initiation, progression, and regulation, this review aims to present a comprehensive analysis of changes in LACTB expression in various cancers, its diverse functional roles in tumorigenesis, the relationship between its structure and function, and its potential as a target for precision cancer medicine.

## 2. Expression Level of the LACTB in Different Cancers

During tumorigenesis, abnormal cell proliferation and survival are fundamental processes, with tumor suppressor genes and oncogenes playing pivotal roles. In most cases, LACTB expression levels are inversely correlated with cancer cell proliferation but exhibit minimal impact on non-tumor cell growth. Reduced LACTB expression frequently correlates with adverse clinical outcomes. However, in certain cancers, such as nasopharyngeal carcinoma and pancreatic adenocarcinoma, LACTB expression is abnormally elevated and strongly associated with poor patient survival rates.

**Figure 1:**
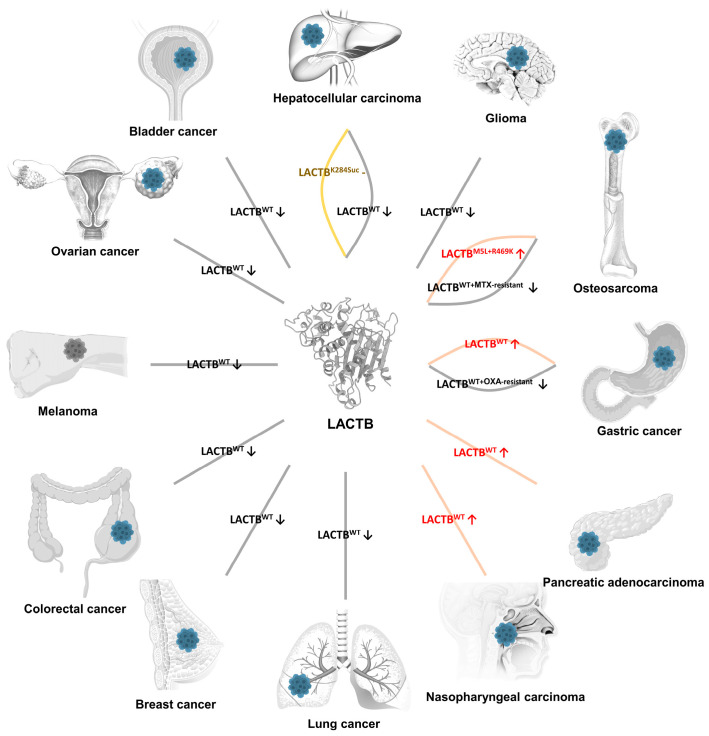
Expression levels of LACTB in various cancer types. In most cancer types, including lung cancer, breast cancer, colorectal cancer, melanoma, ovarian cancer, bladder cancer, hepatocellular carcinoma, glioma, MTX-resistant osteosarcoma, and OXA-resistant gastric cancer, the expression of LACTB^WT^ is significantly downregulated. However, in hepatocellular carcinoma, while the expression levels of LACTB remain unchanged, its function is altered due to post-translational acetylation at the K284 residue. In osteosarcoma, LACTB^M5L+R469K^ demonstrates upregulated expression. Despite these variations, the functional activity of LACTB is generally suppressed in these cancer types, regardless of its expression levels. Conversely, certain cancers such as gastric cancer, pancreatic adenocarcinoma, and nasopharyngeal carcinoma exhibit upregulated LACTB^WT^ expression, which is accompanied by an enhancement of its functional activity. The black arrow represents decreased protein expression level, the red arrow represents increased protein expression level, and the brown short line segment represents unchanged protein expression level. MTX, methotrexate; OXA, oxaliplatin.

Subsequent sections explore the differential expression of LACTB in various cancer types (Figure 1), with the aim to elucidate its roles in tumorigenesis and cancer progression.

### 2.1. Lung Cancer

Lung cancer is among the most prevalent malignant tumors globally and continues to lead cancer-related mortality worldwide, with an estimated 2 million new cases diagnosed annually [10]. Research using data from The Cancer Genome Atlas (TCGA) and Kaplan–Meier Plotter databases indicates that LACTB expression is markedly reduced in lung cancer tissues, accompanied by increased methylation levels on its DNA. The study also shows that elevated LACTB expression is strongly associated with improved prognosis in lung cancer patients and effectively suppresses cancer cell migration and invasion. Additionally, LACTB enhances the antiproliferative effects of docetaxel on cancer cells [11]. Collectively, these findings underscore the critical tumor-suppressive role of LACTB in lung cancer progression.

### 2.2. Breast Cancer

Breast cancer has surpassed lung cancer as the most prevalent cancer worldwide, with its incidence increasing at an annual rate of 0.5% between 2010 and 2019 [12]. Consequently, breast cancer has become a significant global health threat. Research indicates that the expression of LACTB is downregulated in breast cancer, and this downregulation is associated with poor prognosis in breast cancer patients [13]. Moreover, overexpression of LACTB suppresses breast cancer cell proliferation, migration, and invasion, while inducing caspase-independent cell death pathways, thereby inhibiting cancer progression [14,15].

### 2.3. Colorectal Cancer

In the United States, colorectal cancer ranks third in incidence and second in mortality among all cancers [9]. Due to the functional significance of colorectal cancer, research on LACTB in relation to colorectal cancer has become a key focus in LACTB studies. Studies focusing on LACTB regulation in colorectal cancer reveal that microRNAs (miRNAs) such as miR-373-5p and miR-1276 mediate a significant reduction in LACTB expression [16,17]. Furthermore, research has shown that reduced LACTB levels correlate with poor prognosis and advanced clinical stages. Conversely, increased LACTB expression suppresses colorectal cancer onset and progression [18,19,20].

### 2.4. Melanoma

Melanoma is a highly malignant tumor originating from melanocytes in the skin [21]. In melanoma tissues, LACTB expression is markedly downregulated compared to normal skin cells. Research has demonstrated that the overexpression of LACTB effectively suppresses melanoma cell proliferation, migration, and invasion, while promoting apoptosis and inducing G2/M phase cell cycle arrest. These findings reinforce the consistent role of LACTB as a tumor suppressor in melanoma, similar to its function observed in other malignancies [22,23].

### 2.5. Ovarian Cancer

Ovarian cancer is a highly lethal gynecological malignancy and a major cause of death among patients with gynecological tumors [24]. Research indicates that LACTB expression is significantly reduced in ovarian cancer tissues compared to normal ovarian tissues. Notably, re-expression of LACTB has been shown to effectively inhibit the growth of ovarian cancer cells. Additionally, the expression level of LACTB is positively correlated with overall survival in ovarian cancer patients, with higher LACTB expression associated with longer survival times and improved prognosis [25].

### 2.6. Bladder Cancer

Bladder cancer is a prevalent malignancy of the urinary tract, with approximately 613,791 new cases and 220,349 deaths reported globally in 2022 [24]. Research investigating LACTB in bladder cancer revealed that poly(C)-binding protein 1 (PCBP1) suppresses LACTB expression by binding to its mRNA and accelerating its degradation in cancer cells. Moreover, upregulating LACTB induces mitochondrial dysfunction in cancer cells, ultimately triggering ferroptosis, an iron-dependent form of cell death [26].

### 2.7. Hepatocellular Carcinoma

Hepatocellular carcinoma is one of the most common cancers worldwide. In hepatocellular carcinoma cells, LACTB expression and functional regulation are abnormally altered compared to normal tissues. Studies on hepatocellular carcinoma tissue samples reveal significantly reduced LACTB expression compared to normal tissues, a decrease frequently linked to poor prognosis. Overexpression of LACTB in hepatocellular carcinoma cells and patient-derived xenograft models significantly inhibited cell viability, colony formation, and tumor growth. Conversely, LACTB knockout leads to opposing effects [27,28]. In some hepatocellular carcinoma patients, LACTB expression remains unchanged but undergoes succinylation at the K284 site by oxoglutarate carrier 1 (OXCT1). This modification suppresses the proteolytic activity of LACTB, leading to enhanced mitochondrial function and promoting hepatocellular carcinoma cell growth. Furthermore, succinylation at LACTB K284 is also correlated with poor prognosis in hepatocellular carcinoma patients [29]. In summary, proper functioning of LACTB plays a crucial role in suppressing hepatocellular carcinoma progression.

### 2.8. Glioma

Glioma is a diverse class of tumors originating in the central nervous system, specifically from neuroectodermal-derived glial cells, such as astrocytes, oligodendrocytes, and ependymal cells. These tumors are classified into four pathological grades, ranging from Grade I (benign) to Grade IV (glioblastoma), the most malignant form [30]. Emerging evidence indicates that LACTB expression progressively declines with advancing glioma grade. Notably, the expression of LACTB is significantly reduced in glioblastoma, the most aggressive and lethal form of glioma, when compared to normal neural tissues [31]. Further investigations demonstrated that this decreased expression of LACTB in glioblastoma is strongly correlated with poor clinical outcomes. In contrast, the overexpression of LACTB was shown to suppress tumor cell proliferation, invasion, and angiogenesis, suggesting its potential role as a therapeutic target in glioma treatment [32].

### 2.9. Osteosarcoma

Osteosarcoma is a common malignant bone tumor, with incidence peaking at ages 18 and 60 [33]. Detection of LACTB expression in osteosarcoma cells revealed that LACTB is highly expressed in these cells, and its elevated levels are generally associated with poor prognosis [34,35]. Further studies indicated that approximately 92.31% of osteosarcoma patients with high LACTB expression level exhibit two mutations, M5L and R469K, in the protein. These mutations impair the tumor-suppressive function of wild-type LACTB and confer oncogenic-like properties to the protein [36]. In addition, another research group reported that in methotrexate (MTX)-resistant osteosarcoma cells, LACTB expression is relatively reduced, suggesting that downregulation of LACTB may be associated with chemoresistance in osteosarcoma [37].

### 2.10. Gastric Cancer

Gastric cancer is the fifth most common cancer and the third leading cause of cancer-related death worldwide. Risk factors include *helicobacter pylori* infection, age, high salt intake, and low consumption of fruits and vegetables [38]. According to relevant studies, transcriptional analysis of LACTB in gastric cancer tissues and peripheral blood from gastric cancer patients revealed that LACTB transcript 1 is significantly upregulated compared to healthy individuals. In vitro experiments with AGS and HGC-27 cell lines showed that the elevated expression of LACTB transcript 1 promotes the migration and invasion capabilities of gastric cancer cells. Notably, analysis of the two cell lines revealed that in HGC-27, a poorly differentiated cancer cell line, the expression of LACTB protein is, similar to most other cancers, significantly lower than that in normal gastric mucosal tissues [39]. Additionally, studies showed that in oxaliplatin (OXA)-resistant gastric cancer patients and those receiving neoadjuvant chemotherapy (NACT) with OXA plus S-1, LACTB expression is markedly downregulated, which is strongly correlated with poor treatment outcomes [40,41].

### 2.11. Pancreatic Adenocarcinoma

Pancreatic adenocarcinoma, the most common type of pancreatic cancer, accounts for over 90% of malignant pancreatic tumors. As one of the deadliest solid tumors, it is associated with extremely high mortality and low survival rates [42]. Interestingly, research showed that in contrast to its phenotype in most cancers, LACTB mRNA is significantly upregulated in pancreatic adenocarcinoma tissues. Moreover, the immunoreactive score of LACTB protein is markedly higher in pancreatic adenocarcinoma compared to adjacent non-cancerous pancreatic tissue. Elevated LACTB expression in tumor tissues is strongly associated with poor prognosis in pancreatic adenocarcinoma patients [43].

### 2.12. Nasopharyngeal Carcinoma

Nasopharyngeal carcinoma is a malignant tumor with distinct geographical distribution, primarily affecting populations in Southeast Asia and southern China [44]. Research on nasopharyngeal carcinoma has revealed that LACTB expression is significantly elevated in cancer tissues compared to adjacent normal tissues. Elevated LACTB expression correlates with the aggressive behavior of nasopharyngeal carcinoma and reduces overall survival in patients. Notably, high LACTB levels are frequently associated with distant metastasis and treatment failure, emphasizing its potential role in the progression of nasopharyngeal carcinoma [45].

## 3. Mechanistic Roles of LACTB in Cancer Cell Dynamics

The varied expression patterns of LACTB protein in different cancer types highlight its distinct functional roles in tumor biology. In most cases, LACTB acts as a tumor suppressor, inhibiting cancer progression. However, in specific cancers, LACTB exhibits oncogenic properties, significantly contributing to malignancy. In the following sections, we provide an in-depth discussion and analysis of the regulatory mechanisms controlling LACTB expression in cancer and the pathways through which it modulates cancer dynamics.

### 3.1. Regulatory Mechanisms of LACTB Expression

**Figure 2:**
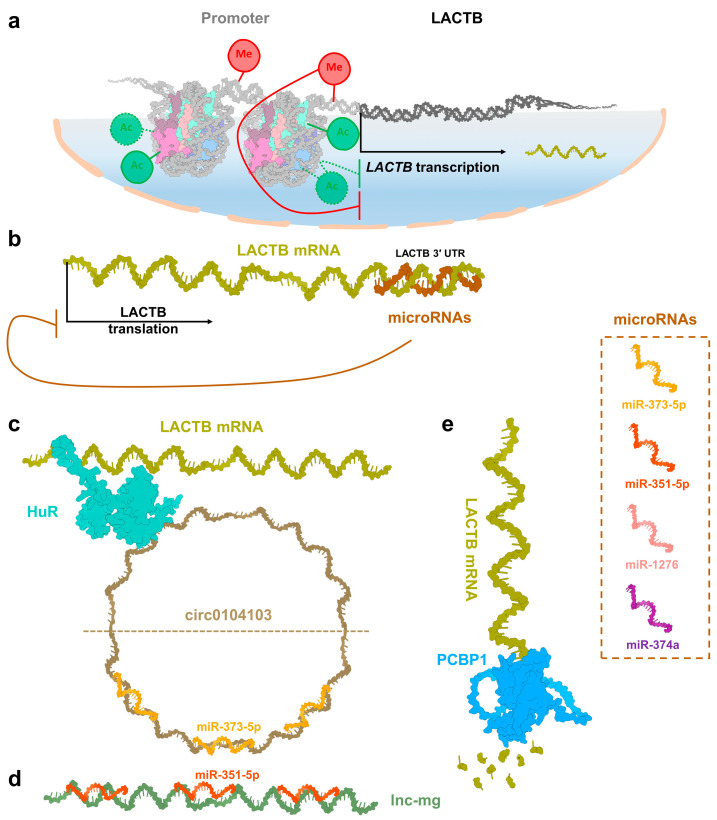
LACTB expression regulatory mechanisms. Regulatory mechanisms controlling LACTB expression at the transcriptional, post-transcriptional, and translational levels. (**a**) In the nucleus, hypermethylation of the *LACTB* promoter region and hypoacetylation of histones suppress *LACTB* transcription, resulting in reduced protein expression. (**b**) At the post-transcriptional level, miRNAs inhibit LACTB mRNA translation by binding to the 3′-UTR of its mRNA, thereby downregulating its expression. Specific miRNAs involved in this regulation include miR-351-5p, miR-1276, miR-374a, and miR-373-5p. (**c**) In colorectal cancer cells, circular RNA circ0104103, a 694 bp circular RNA derived from exons 3 to 5 of the *LACTB* gene, enhances LACTB expression through two mechanisms: it acts as a molecular sponge to sequester miR-373-5p, freeing LACTB mRNA, and recruits HuR to stabilize LACTB mRNA, collectively promoting LACTB translation. (**d**) In muscle cells, the lnc-mg competitively interacts with miR-351-5p as a molecular sponge, thereby releasing LACTB mRNA and increasing its expression. (**e**) The PCBP1 protein negatively regulates LACTB expression by degrading its mRNA, further reducing LACTB protein levels. HuR, human antigen R; lnc-mg, myogenesis-associated long noncoding RNA; PCBP1, poly(C)-binding protein 1; 3′-UTR, 3′ untranslated region; miRNAs, microRNAs.

Modifications in promoter regions, such as single nucleotide polymorphisms (SNPs), DNA methylation, and histone modifications, significantly affect protein expression. Such regulatory changes profoundly impact protein expression and are closely associated with cancer development and progression [46,47,48]. In nasopharyngeal carcinoma cells, LACTB expression is distinctly regulated by DNA methylation. In the low-metastatic parental nasopharyngeal carcinoma cell line CNE-2, the DNA in the 5′ promoter region of *LACTB* is highly methylated, leading to suppressed mRNA transcription of *LACTB*. Conversely, in the highly metastatic S18 cell line, which is derived from CNE-2, the methylation level at this site is reduced, resulting in upregulated *LACTB* transcription. Elevated LACTB expression in S18 cells has been associated with enhanced nasopharyngeal carcinoma progression (Figure 2a) [45]. Beyond nasopharyngeal carcinoma, similar regulatory mechanisms are observed in colorectal cancer. Hypermethylation of 31 CpG sites within the *LACTB* promoter region (−31 to +235 bp) has been identified, contributing to reduced LACTB expression. Treatment with the DNA methyltransferase inhibitor 5-Aza-dC restores LACTB expression in colorectal cancer cells, demonstrating the reversibility of this epigenetic modification. Additionally, histone modifications also play a crucial role in LACTB expression regulation. The histone deacetylase inhibitor TSA has been shown to modulate LACTB expression by targeting histone acetylation patterns in its promoter region. Hypoacetylation of histones, particularly histone H3, in the *LACTB* promoter region is strongly associated with reduced LACTB expression (Figure 2a) [19]. These findings highlight the pivotal roles of promoter methylation and histone hypoacetylation in the regulation of LACTB expression.

In addition to transcriptional regulation, research on LACTB expression in various cancers has revealed that LACTB mRNA translation is also finely regulated within cancer tissues. MicroRNAs, a significant class of non-coding RNAs typically comprising 18 to 25 nucleotides, primarily regulate transcription and translation by binding to the 3′ untranslated region (3′-UTR) of target mRNAs, thus inhibiting translation or promoting mRNA degradation [49]. Recent studies identified miRNAs such as miR-373-5p and miR-1276 in colorectal cancer and miR-374a in breast cancer, which bind to the 3′-UTR of LACTB mRNA, suppressing its translation, reducing protein levels, and impairing its tumor-suppressive functions (Figure 2b) [13,16,17]. Interestingly, these miRNA-mediated regulatory effects on LACTB mRNA translation are counterbalanced by other molecular factors. For example, in normal colorectal tissues, circ0104103, a 694 bp circular RNA derived from *LACTB* exons 3–5, sponges miR-373-5p to stabilize LACTB expression (Figure 2c) [17]. Additionally, in the CRC cell line HCT116, circ0104103 directly interacts with human antigen R (HuR), a key RNA-binding protein. Through this interaction, HuR is recruited to bind to LACTB mRNA, stabilizing it and mitigating the downregulation of LACTB expression (Figure 2c) [17]. Similar regulatory mechanisms are also observed in non-cancerous cells. For example, in muscle tissue, miR-351-5p targets the 3′-UTR of LACTB mRNA, suppressing its expression and affecting myogenesis. However, a myogenesis-associated long noncoding RNA (lnc-mg) also functions as a molecular sponge for miR-351-5p, neutralizing its inhibitory effect on LACTB mRNA translation and promoting myogenic processes (Figure 2d) [7]. Beyond miRNA-mediated alterations, other molecules, such as protein enzymes, also influence LACTB expression levels. For instance, in bladder cancer, PCBP1 directly promotes LACTB mRNA degradation, thereby regulating its translation and suppressing its tumor-suppressive function (Figure 2e) [26].

### 3.2. Impact of LACTB on Mitochondrial Activity

Mitochondria, as essential metabolic hubs within the human body, are primarily responsible for energy production, intracellular metabolic regulation, and signal transduction [50]. Mitochondrial dysregulation plays a crucial role in cancer development [51]. As the only double-membraned organelles in mammalian cells, mitochondria rely on compartmentalization to maintain their fundamental functions. This compartmentalized structure offers separate environments for distinct metabolic processes, effectively preventing interference among various metabolic pathways and confining damaging agents, such as reactive oxygen species (ROS), to specific regions, thus protecting other cellular components from potential damage [52]. Moreover, the stability of mitochondrial cristae morphology is vital for proper mitochondrial function. Recent research showed that mitochondrial respiratory chain complexes and supercomplexes require membranes with precise curvature to stabilize their assembly [53,54]. For instance, the symmetrical arrangement of complex V at the cristae tips necessitates a highly curved membrane environment [55,56], highlighting the importance of maintaining stable cristae morphology to support the structural demands of these complexes. Additionally, stable cristae morphology is crucial for preserving mitochondrial membrane potential (Δψm), ensuring consistent ATP production for various biological activities [57].

Mitochondria are also the primary site of lipid metabolism, synthesizing essential phospholipids such as phosphatidylethanolamine (PE), phosphatidylglycerol (PG), cardiolipin (CL), and the redox-active lipid coenzyme Q (CoQ, ubiquinone) [58,59]. Recently, research demonstrated that mitochondrial phospholipids significantly influence mitochondrial morphology, cristae formation, membrane protein function, mitophagy, and cell death [60]. PE, in particular, is synthesized de novo from phosphatidylserine (PS) by phosphatidylserine decarboxylase (PISD) on the inner mitochondrial membrane. This PE is then transported to the endoplasmic reticulum, where it is further converted into phosphatidylcholine (PC), a major component of cell membranes [61]. Research indicates that abnormal PISD function often results in mitochondrial dysfunction and structural abnormalities [62,63], underscoring the critical role of de novo PE synthesis in mitochondrial compartmentalization.

**Figure 3:**
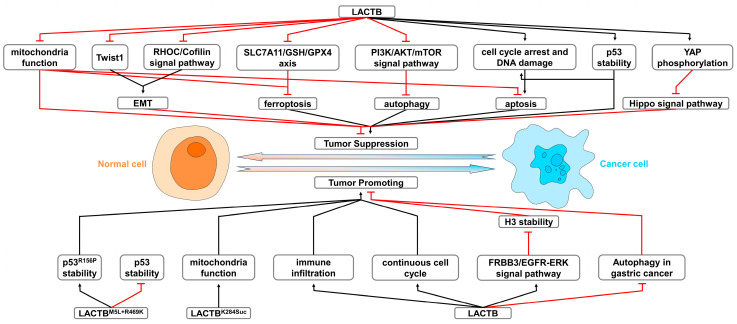
Tumor suppression and promoting mechanisms of LACTB. LACTB exhibits dual roles in cancer biology, functioning as both a tumor suppressor and promoter depending on the context and cancer type. As a tumor suppressor, LACTB expression influences mitochondrial structure and function, stabilizes p53 protein, activates cell death pathways such as ferroptosis, autophagy, and apoptosis, inhibits EMT, and suppresses the Hippo signaling pathway, thereby suppressing tumor progression. Conversely, in cancers such as pancreatic adenocarcinoma, gastric cancer, and nasopharyngeal carcinoma, LACTB promotes tumorigenesis by destabilizing H3 histone proteins, facilitating cell cycle progression, and enhancing immune infiltration within the tumor microenvironment. Additionally, post-translational modifications and mutations of LACTB contribute to its dual function, with succinylation at K284 promoting cancer progression through enhanced mitochondrial function, while the LACTB^M5L+R469K^ mutant stabilizes the p53^R156P^ protein and accelerates the degradation of wild-type p53, exerting tumor-suppressive effects. These findings highlight the complex, context-dependent roles of LACTB in cancer, emphasizing its potential as a therapeutic target. EMT, epithelial-mesenchymal transition. The red lines indicate the inhibitory effects on downstream signaling pathways, while the black arrows represent the promoting effects.

LACTB is a polymeric protein that forms elongated filamentous structures, interacting with membrane components via its terminal and central regions. The sixth loop region of LACTB, spanning residues K255 to K284, has been identified as crucial for its interactions with membranes. Within this region, a charged and hydrophobic motif (259KNxFxKFK266) along with a cluster of six basic side chains (274KxRxxKxxKKK284) are primarily responsible for mediating these interactions [3]. Such interactions play a fundamental role in maintaining mitochondrial compartmentalization and cristae morphology [2,3]. Moreover, studies on LACTB in cancer revealed that LACTB expression and its enzymatic activity effectively suppress PISD protein levels, thereby limiting the excessive synthesis and accumulation of PE in cancer cell mitochondria. This regulation impacts mitochondrial compartmentalization and cristae morphology from a distinct perspective [15]. Research on LACTB in cancers such as breast cancer, bladder cancer, gastric cancer, and melanoma has consistently demonstrated its profound impact on mitochondrial function (Figure 3), inducing mitochondrial depolarization in cancer cells and significantly affecting mitochondrial respiration, Δψm, ATP production, and ROS-related cellular damage [14,15,22,26,40]. Additionally, a study in hepatocellular carcinoma revealed that succinylation at the K284 residue of LACTB enhances mitochondrial function without altering its protein levels (Figure 3). This functional enhancement is hypothesized to stem from altered interactions between LACTB and mitochondrial membranes, combined with changes in enzymatic activity, which together affect mitochondrial lipid metabolism [3,29].

### 3.3. Impact of LACTB on Epithelial–Mesenchymal Transition

Epithelial–mesenchymal transition (EMT) processing in cancer cells is a key physiological process by which cancer cells transition from an epithelial to a mesenchymal state, acquiring enhanced migratory, invasive, and stem-like characteristics [64]. Aberrant LACTB expression in cancer cells is closely associated with EMT regulation. In various cancers, including gastric, colorectal, lung, glioma, and ovarian cancers, abnormal expression of LACTB leads to downregulation of the epithelial marker E-cadherin at mRNA and protein levels, while concurrently upregulating mesenchymal markers such as N-cadherin, vimentin, snail, slug, and matrix metalloproteinases MMP2 and MMP9 [11,16,20,25,31,39]. This shift enhances the migratory and invasive potential of cancer cells. Modulating LACTB expression in various cancer cell types can effectively reverse these marker expressions, thereby reducing cellular migration and invasion. Further research in colorectal cancer has shown that LACTB regulates the expression of Twist1, a transcription factor for mesenchymal stem cell markers, in a PI3K pathway-dependent manner, inhibiting EMT progression (Figure 3) [20]. Beyond directly modulating epithelial and mesenchymal marker expression, elevated LACTB expression in glioblastoma was shown to reduce Rho-related GTP-binding protein RhoC (RHOC) protein levels, suppressing the RHOC/Cofilin signaling pathway (Figure 3) [32]. This downregulation affects actin and myosin polymerization, enabling control of cancer cell morphology and influencing EMT progression [32]. The role of LACTB in regulating EMT in various cancers thus underscores its broader functional significance in tumor progression.

### 3.4. Impact of LACTB on Cell Death Pathways

Cell death represents the irreversible cessation of cellular function and marks the end of a cell life cycle. It is essential for maintaining tissue integrity and function, playing vital roles in tissue homeostasis and host defense [65]. Furthermore, cell death pathways significantly influence cancer initiation and progression [66,67]. Recent research identified LACTB as a critical regulator of cancer cell death pathways, predominantly acting as a tumor suppressor via mechanisms such as ferroptosis, autophagy, and apoptosis. In liver cancer, LACTB enhances ferroptosis by inhibiting the cystine/glutamate transporter SLC7A11/GSH/glutathione peroxidase 4 (GPX4) antioxidant pathway and activating nuclear receptor coactivator 4 (NCOA4)-mediated ferritinophagy via a p53-dependent process (Figure 3). This regulation increases iron-dependent lipid peroxidation, thereby limiting tumor growth [28]. Similarly, in bladder cancer, LACTB modulates ferroptosis and mitochondrial function by inducing erastin-mediated ferroptosis, which exacerbates mitochondrial dysfunction and ROS production (Figure 3), counteracting the ferroptosis-inhibitory effects of PCBP1. PCBP1 protects mitochondrial integrity by destabilizing LACTB mRNA, thereby reducing ferroptosis in bladder cancer cells [26].

The role of LACTB in autophagy regulation further underscores its dual influence as both a tumor suppressor and, in certain contexts, an oncogene. In gastric cancer, LACTB suppresses autophagy, thereby modulating immune resistance and cancer stemness (Figure 3), emphasizing its significance in precision oncology [39]. A similar phenomenon is observed in OXA-resistant gastric cancer cells (MGC-803/OXA), where LACTB overexpression inhibits autophagy. This is evidenced by increased p62 protein levels, a reduced LC3II/I ratio, and decreased Beclin-1 expression, further supporting its autophagy-suppressive role in this context [40]. Conversely, in colorectal cancer, LACTB activates autophagy by inhibiting the PI3K/AKT/mTOR pathway then suppressing cell proliferation (Figure 3) [20].

LACTB regulates apoptosis in various cancers (Figure 3). In breast cancer, LACTB induces caspase-independent apoptosis by generating ROS, causing DNA damage, and activating pro-apoptotic proteins such as Puma, Bim, and Bax [14]. In melanoma, LACTB promotes apoptosis and tumor suppression by modulating mitochondrial lipid metabolism and stabilizing p53, thereby upregulating pro-apoptotic genes like p21, Bax, and Bid [23]. In ovarian cancer, overexpressed LACTB drives cancer cells to accumulate in the G1 phase, thereby promoting apoptosis (Figure 3) [25]. Interestingly, in OXA-resistant gastric cancer cells, LACTB overexpression, despite suppressing autophagy, effectively induces apoptosis. This is achieved by causing genomic DNA damage, disrupting mitochondrial function, reducing glucose uptake, and inhibiting ATP synthesis (Figure 3) [40]. Collectively, these studies establish LACTB as a versatile regulator of cancer cell death pathways. Through its roles in ferroptosis, autophagy, and apoptosis, LACTB demonstrates a multifaceted approach to modulating cell death in diverse cancer types. These findings emphasize its therapeutic potential as a target for cancer treatment, offering new avenues for precision oncology.

### 3.5. Impact of LACTB on p53-Dependent Cancer Suppression

The p53 protein is a well-established tumor suppressor that regulates the cell cycle, promotes cell death, and maintains genomic stability [68]. Research has shown that abnormal expression of LACTB is associated with p53 expression level and cellular localization in colorectal cancer, melanoma, liver cancer, and osteosarcoma. In colorectal cancer cells, LACTB overexpression prolongs the half-life of p53 without altering p53 mRNA levels (Figure 3). By interacting with the C-terminal domain of p53, LACTB inhibits the binding of the E3 ubiquitin–protein ligase Mdm2 (MDM2) to p53, protecting it from degradation [19,69]. This protection upregulates downstream targets of p53, such as the cell cycle regulator p21 and apoptosis-related proteins Bax and cleaved caspase-3, thereby suppressing cancer progression [17,19]. Similarly, in melanoma cells, elevated LACTB expression enhances the expression of p21, Bax, Bid, Padd1, and Sival, exerting tumor-suppressive effects through p53 signaling [23]. In liver cancer cells, LACTB overexpression also prolongs the half-life and nuclear localization of p53; however, in contrast to colorectal cancer, stabilized p53 in liver cancer promotes ferroptosis by directly binding to the *heat shock protein family A (Hsp70) member 8* (*HSPA8*) promoter and reducing HSPA8 expression [28]. Distinctly, in osteosarcoma cells, LACTB is highly expressed in its mutant form LACTB^M5L+R469K^, which binds directly to proteasome subunit beta-type 7 (PSMB7), promoting p53 degradation while inhibiting the nuclear export of the p53^R156P^ variant, thus exhibiting oncogenic properties (Figure 3) [36]. In summary, LACTB modulates p53 pathways through diverse mechanisms, exerting distinct effects on cancer progression in different cancers.

### 3.6. Impact of LACTB on the Hippo Pathway

The Hippo pathway plays a vital role in regulating organ morphology, tissue homeostasis, and the initiation and progression of various cancers. During tumorigenesis, dysregulation of the Hippo pathway leads to excessive activation of downstream effectors, driving uncontrolled cell proliferation and tumor growth [70]. Yes-associated protein (YAP), a key transcriptional coactivator in the Hippo pathway, primarily binds with transcriptional enhancer factor TEF (TEAD) proteins in the nucleus to activate downstream genes involved in cell proliferation and apoptosis [71]. YAP requires dephosphorylation to function effectively in the nucleus [72]. In melanoma cells, LACTB loss allows the catalytic subunit alpha of protein phosphatase-1 (PP1A) to effectively dephosphorylate YAP [73], leading to activation of the Hippo pathway and promoting tumorigenesis. Conversely, exogenous expressed LACTB directly interacts with PP1A, inhibiting its dephosphorylation effect on YAP. This inhibition prevents YAP phosphorylation, retaining it in the cytoplasm and obstructing subsequent oncogenic signaling, thus suppressing cancer progression without altering YAP expression levels (Figure 3) [22].

### 3.7. Impact of LACTB on Histone and Its Post-Translational Modifications

Histones and their post-translational modifications are critical in numerous biological processes, including chromatin structural stability, gene expression, cell differentiation, autophagy, and cancer development [74]. As key members of the histone family, histones H3 and H4 form an octamer that provides a scaffold for DNA wrapping, directly influencing chromatin conformation and function [75]. Mutations and modifications in histone H3 can disrupt chromatin compaction, increase instability, and drive cancer initiation and progression [76,77,78]. In nasopharyngeal carcinoma cells, LACTB upregulation increases receptor tyrosine protein kinase erbB-3 (ERBB3) expression, which dimerizes with epidermal growth factor receptor (EGFR) to activate downstream mitogen-activated protein kinase (MAPK) and RAC serine/threonine protein kinase (AKT) signaling pathways. The activation of these pathways decreases histone H3 stability and acetylation levels, while promoting its ubiquitination and degradation. Reduced histone H3 stability disrupts chromatin modification patterns and impairs normal gene regulatory functions, leading to chromatin collapse, increased genomic instability, and ultimately promoting metastasis in nasopharyngeal carcinoma cells (Figure 3) [45].

### 3.8. Impact of LACTB on Immune Infiltration

Tumor immune infiltration plays a dual role in cancer progression. On the one hand, immune cells such as tumor-associated macrophages (TAMs), myeloid-derived suppressor cells (MDSCs), and regulatory T cells (Tregs) secrete factors like transforming growth factor beta (TGF-β) and interleukin-10 (IL-10), promoting angiogenesis, immune suppression, and immune evasion, thereby facilitating tumor growth and metastasis [79,80]. Conversely, cytotoxic T lymphocytes (CTLs), natural killer (NK) cells, and dendritic cells (DCs) release cytokines such as interferon gamma (IFN-γ) and interferon alpha (TNF-α) to identify and eliminate tumor cells, thereby inhibiting tumor growth [81,82,83]. In pancreatic adenocarcinoma, patients with elevated LACTB expression generally exhibit greater immune infiltration, as evidenced by positive associations with 28 distinct immune cell types. However, these patients often experience increased treatment challenges and poor prognosis, indicating that the immune infiltration associated with high LACTB expression may promote tumor progression in pancreatic adenocarcinoma (Figure 3) [43].

### 3.9. Impact of LACTB on Cell Cycle Arrest

The cell cycle is rigorously controlled in normal cell development, with checkpoints ensuring accurate DNA replication and segregation. In cancer cells, this control is frequently disrupted, leading to unchecked cell cycle progression, compromised DNA damage checkpoints, and dysregulated cell cycle exit mechanisms, resulting in increased proliferation and genomic instability [84]. Recent studies revealed a complex role for LACTB in regulating the cell cycle and influencing tumor growth in different cancers. In pancreatic adenocarcinoma, LACTB is highly expressed and correlates with elevated levels of cell cycle-related gene. This suggests that LACTB appears to facilitate continuous cell cycle progression and unchecked cell cycle progression, thus enabling sustained proliferation and potentially aiding cancer development rather than suppressing it (Figure 3) [43]. In breast cancer, however, LACTB expression effectively induces G1 phase arrest (Figure 3), which is linked to increased mitochondrial ROS production, leading to caspase-independent cell death [14]. Similarly, in ovarian cancer, restoring LACTB expression in cancer cells leads to cell cycle arrest at G1 (Figure 3), which suppresses cell proliferation, demonstrating LACTB as a tumor suppressor in this context [25]. In melanoma, delivery of LACTB via gene therapy showed promising results in both in vitro and in vivo models, where LACTB successfully inhibited cell cycle progression, induced G2/M cell cycle arrest, and enhanced apoptosis, further establishing its role in cell cycle regulation (Figure 3) [23]. Additionally, in colon cancer, LACTB was shown to be suppressed by miR-1276, but when overexpressed, it exerted strong anti-proliferative effects by inducing cell cycle arrest (Figure 3) [16]. Collectively, these findings underscore the dual role of LACTB in cancer biology, as it can either promote cell cycle arrest to suppress tumor growth or, in certain contexts, support continuous cell cycle progression to support cancer development.

## 4. Current Research on the Structure of LACTB and Its Association with Cancer

**Figure 4:**
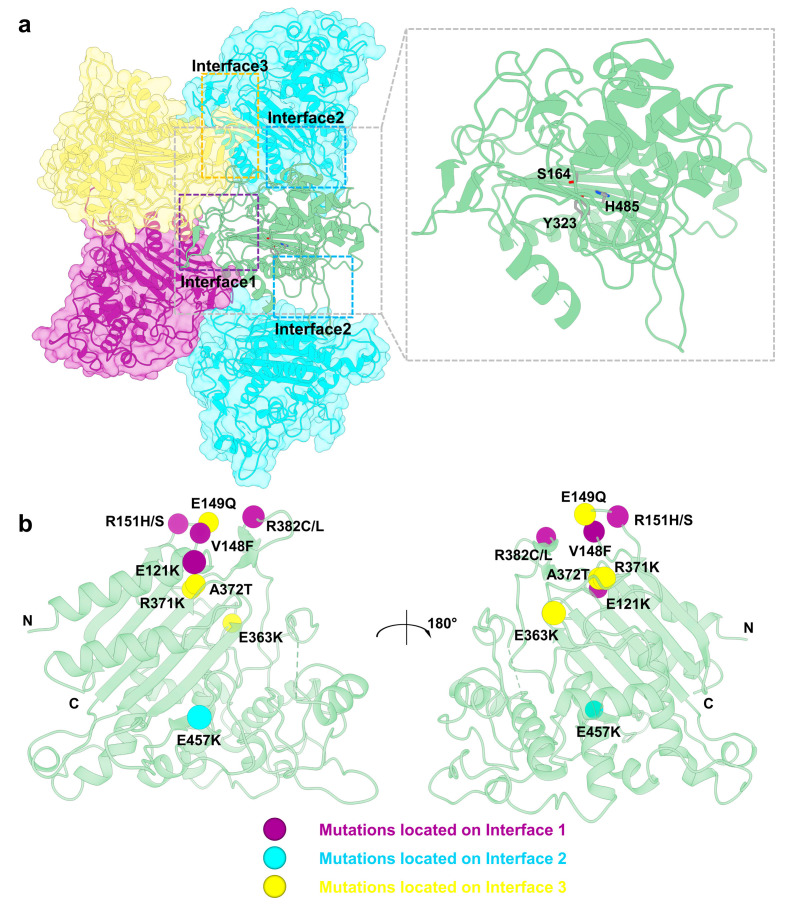
Structural organization and cancer-related mutations of LACTB. (**a**) LACTB assembles into long filaments through three primary interaction interfaces, termed interface 1, interface 2, and interface 3. On the left, a segment of the LACTB filament comprising five subunits is shown, with each individual LACTB monomer forming four interaction interfaces with neighboring subunits. These interactions are grouped into the three main interfaces: interface 1 (purple), interface 2 (orange), and interface 3 (blue). A single LACTB monomer is highlighted with a gray box and magnified on the right to show its structure in detail. Key catalytic residues are labeled to emphasize their functional relevance. (**b**) Cancer-related mutations in LACTB are mapped onto a single LACTB structure. Mutations located within interface 1 are marked with purple dots, those in interface 2 with orange dots, and those in interface 3 with blue dots. All structural models were generated using ChimeraX-1.8 [85] software based on the Protein Data Bank (PDB) entry 7V1Z.

LACTB is homologous to prokaryotic PBP-βLs family proteins, sharing a common catalytic SXXK motif (where X is any amino acid) in its sequence [2]. Structurally, similar to other family members, LACTB contains a stable PBP-βLs domain, with the SXXK motif centrally positioned within the monomer, facilitating substrate hydrolysis (Figure 4a) [1,2,3]. Structural studies revealed that LACTB primarily recognizes and hydrolyzes peptide bonds following aspartic acid residues [1]. In addition to the conserved PBP-βLs domain, LACTB contains a unique middle region (E224-Q289) absent in other PBP-βLs family proteins. This middle region is located directly above the catalytic site, with structural studies identifying a flexible loop (K243-Q289) in this area. The lack of a stable model suggests potential specialized functions [1,3]. Enzyme activity assays on the LACTB deletion mutant, LACTB^ΔE224-Q289^ demonstrate a loss of hydrolytic activity against the standard substrate Ac-YVAD-AMC, emphasizing the critical role of this domain in substrate hydrolysis [1]. Additionally, studies on this region revealed that the middle region is essential for the interaction between LACTB and mitochondrial membrane components [3].

The primary distinction between LACTB and prokaryotic PBP-βLs family proteins is the unique ability to form a DNA-like double-helical filament structure (Figure 4a) [1,3], which plays a role in maintaining mitochondrial compartmentalization [2,3]. In the formation of this filament, LACTB PBP-βLs domain acts as the structural scaffold, while the middle region has a relatively minor influence. Studies on the filament structure reveal that each LACTB monomer directly interacts with four neighboring monomers, promoting filament assembly. Based on distinct interaction interfaces, three interaction surfaces have been identified around each LACTB protein, referred to as interfaces 1, 2, and 3. Interface 2 mediates interactions between adjacent LACTB proteins within the same chain, whereas interfaces 1 and 3 facilitate interactions between two different chains [1]. Further research indicated that the filament structure of LACTB is linked to its substrate hydrolysis efficiency [1], interactions between single chains enhance catalytic rates [1,3], while interactions between monomers within a single chain affect the Km value of LACTB [1]. Additionally, study on LACTB filament structure suggests that both the apex and lateral sides of the LACTB filament are capable of binding to membrane components [3]. Overall, the filament structure of LACTB significantly enhances substrate hydrolysis and provides the structural foundation for its role in mitochondrial compartmentalization.

Research on LACTB functions has shown that its enzymatic activity is essential for its tumor-suppressive role [15]. Furthermore, the influence of LACTB on mitochondrial morphology is crucial for its tumor-suppressive functions [15]. The maintenance of the filament structure directly impacts both enzymatic activity and the regulation of mitochondrial cristae morphology, highlighting the important role of LACTB filament in cancer. To date, over 200 variants of the LACTB gene have been identified, with approximately 30% associated with disease. Among these pathogenic variants, 70% are missense mutations linked to various cancers [86,87,88]. Mapping these mutations onto the filament structure reveals multiple hotspot mutations at interaction sites within the double-helical structure, associated with different cancer types (Figure 4b, Table 1) [1,3]. Certain mutations like E121K, V148F, E149Q, R151S, R371K, R382L, R382C, and E457K have been validated as critical for the LACTB filament-forming ability (Table 1) [1]. These findings suggest a potential relationship between the ability to polymerize into filaments and pathogenicity.

## 5. Investigation of LACTB as a Target for Precision Medicine in Cancer

As mentioned above, abnormal expression of LACTB in various cancers is strongly associated with cancer progression and poor postoperative prognosis. Studies have shown that modulating LACTB expression in cancer cells can positively impact the EMT process [11,16,20,25,31,32,39], encourage cell cycle arrest [14,16,23,25,43], and induce cancer cell death via autophagy [20,39,40], apoptosis [14,23,25], and ferroptosis [26,28]. These effects collectively inhibit tumor progression and significantly reduce tumor growth in xenograft mouse models. Therapeutic research focused on LACTB modulation further indicates that LACTB modulation enhances drug sensitivity in both cancer cells and xenograft models [11,28,36]. For instance, the β-lactamase inhibitor clavulanate potassium was shown to inhibit osteosarcoma proliferation by binding to and blocking the LACTB^M5L+R469K^ mutant, thereby increasing cancer cell sensitivity to cisplatin [36]. Similarly, LACTB overexpression was associated with increased sensitivity to docetaxel in lung cancer cells and enhanced the antitumor efficacy of lenvatinib in liver cancer samples [11,28]. These findings support the potential for LACTB modulation as a novel approach in cancer therapy development. Given its role in cancer progression, treatment response, and prognosis, LACTB emerges as a promising target in precision oncology.

Significant progress has been achieved in targeting LACTB for applications in precision medicine. Notably, in melanoma, a cancer type where LACTB is significantly downregulated and associated with poorer patient outcomes, restoring LACTB expression has emerged as a promising therapeutic approach due to its role in inducing apoptosis and modulating tumor growth [22,23]. Recent advancements have introduced a novel nonviral delivery system designed for targeted gene therapy. This system, termed iDPP, is constructed through the self-assembly of components including the targeting peptide C18-PEG-iRGD (iRGD), the cationic lipid N-[1-(2,3-dioleoyloxy) propyl]-N,N,N-trimethylammonium chloride (DOTAP), and monomethoxy poly(ethylene glycol)-poly(D,L-lactide) (MPEG-PDLLA). The iDPP/LACTB nanocomplex has been specifically employed for the delivery of LACTB gene therapy into melanoma cells. Experimental studies have demonstrated that this nanocomplex achieves high transfection efficiency and exhibits significant antitumor activity in both in vitro and in vivo models. Mechanistically, this nanocomplex activated the p53 signaling pathway, leading to enhanced expression of genes involved in cell cycle arrest and apoptosis, thereby reinforcing its tumor-suppressive properties [23]. Moreover, the iDPP system outperformed traditional viral vectors in terms of safety and biocompatibility, highlighting its potential as a promising strategy for further exploration of LACTB as a therapeutic target in precision medicine.

## 6. Conclusions and Perspectives

LACTB protein has emerged as a critical factor in cancer progression and therapy due to its multifaceted biological functions and diverse regulatory mechanisms. Its differential expression in various cancer types underscores its dual role, functioning as a tumor suppressor in some malignancies while exhibiting oncogenic potential in others [11,13,14,15,16,17,18,19,20,22,23,25,26,27,28,29,31,32,34,35,36,37,39,40,41,43,45]. These opposing effects are mediated through complex interactions within key cellular pathways, such as those involving mitochondrial dynamics [2,3], lipid metabolism [15], cell cycle regulation, and the EMT process [11,16,20,25,31,32,39]. Additionally, the influence of LACTB on essential signaling pathways, including the p53 and Hippo pathways [19,22,28,36,69], contributes to its modulation of cancer cell behavior, impacting processes like apoptosis [14,23,25], autophagy [20,39,40], and ferroptosis [26,28]. This tissue-specific variability in function emphasizes the need for a nuanced understanding of the important role of LACTB, which may inform the development of targeted cancer therapies tailored to distinct molecular landscapes.

At the molecular level, the structural domains of LACTB, including its PBP-βLs domain and a unique middle region, are fundamental to its biological activities. The ability of the protein to form filamentous structures within the mitochondrial matrix is central to its functions, affecting its enzymatic capabilities and interactions with mitochondrial membranes [1,3]. Mutations in the LACTB gene, particularly those disrupting filament formation, have been implicated in various cancers, correlating with either diminished tumor-suppressive functions or enhanced oncogenic activity depending on the context [1,3]. Insights into the structure–function relationship of LACTB suggest that targeting its polymerization ability or enzymatic properties may provide innovative therapeutic options for malignancies characterized by dysregulated LACTB expression.

The potential of LACTB as a target in precision medicine is exemplified by advances in therapeutic strategies modulating its activity. Manipulating LACTB expression has been shown to enhance drug sensitivity in cancer cells, thereby serving as a complementary approach to conventional treatments [11,28,36]. For instance, β-lactamase inhibitors like clavulanate potassium, when combined with cisplatin, have improved therapeutic efficacy in osteosarcoma by targeting LACTB mutants [36]. Overexpression of LACTB has also been found to enhance the effects of docetaxel in lung cancer and lenvatinib in liver cancer [11,28]. Moreover, innovative delivery systems such as the iDPP/LACTB nanocomplex have demonstrated high efficiency in gene delivery, substantial anti-tumor activity, and superior safety profiles in preclinical studies [23]. These developments support the potential of LACTB as a promising therapeutic target and provide a foundation for further exploration in personalized cancer treatment.

In summary, leveraging LACTB for cancer therapy could revolutionize oncology by offering targeted, patient-specific treatment strategies. Future research should focus on deepening our understanding of the mechanisms underpinning the dual roles of LACTB in tumor suppression and promotion, as well as the structural attributes that facilitate these functions. By elucidating these aspects and their interactions with major cellular networks, researchers may discover new therapeutic approaches that exploit the biological potential of LACTB. As a critical modulator of both cancer progression and treatment response, LACTB represents a promising target in precision medicine, paving the way for more effective and individualized cancer care.

## Figures and Tables

**Table 1:** Human LACTB mutations associated with cancer.

Mutation Site	Interface	Related Cancer	References
E121K	Interface 1	Breast	[1,3]
V148F	Interface 1	Kidney	[1,3]
E149Q	Interface 3	Esophagus	[1,3]
R151S, R151H	Interface 1	Uterus	[1,3]
E363K	Interface 3	Pancreatic	[3]
R371K	Interface 3	Lung	[1,3]
A372T	Interface 3	Uterus	[3]
R382L, R382C	Interface 1	Oral, uterus	[1,3]
E457K	Interface 2	Bladder	[1,3]

## Data Availability

No new data were created or analyzed in this study. Data sharing is not applicable to this article.
